# Supplementary material for: Operando neutron diffraction reveals mechanisms for controlled strain evolution in 3D printing
Source: Nat Commun. 2023 Aug 16;14:4950. doi: 10.1038/s41467-023-40456-x (PMC10432395; doi:10.1038/s41467-023-40456-x)
Supplement: Supplementary file 1 — Supplementary Information [file 41467_2023_40456_MOESM1_ESM.pdf]

Supplementary Info for

Operando neutron diffraction reveals mechanisms for controlled strain evolution in 3D printing

A. Plotkowski[1*], K. Saleeby[2], C. M. Fancher[1], J. Haley[3], G. Madireddy[2], K. An[4], R. Kannan[2], T. Feldhausen[2], Y. Lee[5], D. Yu[4], C. Leach[3], J. Vaughan[2], S. S. Babu[2, 6]

[1]Materials Science and Technology Division, Oak Ridge National Laboratory

[2]Manufacturing Science Division, Oak Ridge National Laboratory

[3]Electrification and Energy Infrastructure Division, Oak Ridge National Laboratory

[4]Neutron Scattering Division, Oak Ridge National Laboratory

[5]Computational Sciences and Engineering Division, Oak Ridge National Laboratory

[6]Department of Mechanical, Aerospace, and Biomedical Engineering, The University of Tennessee

*Correspondence to: plotkowskiaj@ornl.gov

Notice: This manuscript has been authored by UT-Battelle, LLC, under contract DE-AC05-00OR22725 with the US Department of Energy (DOE). The US government retains and the publisher, by accepting the article for publication, acknowledges that the US government retains a nonexclusive, paid-up, irrevocable, worldwide license to publish or reproduce the published form of this manuscript, or allow others to do so, for US government purposes. DOE will provide public access to these results of federally sponsored research in accordance with the DOE Public Access Plan (http://energy.gov/downloads/doe-public-access-plan).

Supplementary Note 1: Materials and Methods

<u>Additive Manufacturing Materials and Methods</u>

A Tormach ZA6 6 degree-of-freedom robotic arm was integrated with a Tormach PCNC 440 CNC machining center and a Lincoln Electric R450 Welder to form a hybrid (metal additive and subtractive) processing center. This combined system was designed to fit within physical, electrical, and neutron diffraction constraints of the VULCAN beamline at Oak Ridge National Lab's Spallation Neutron Source. A diagram of the complete system is displayed in Supplementary Figure S.

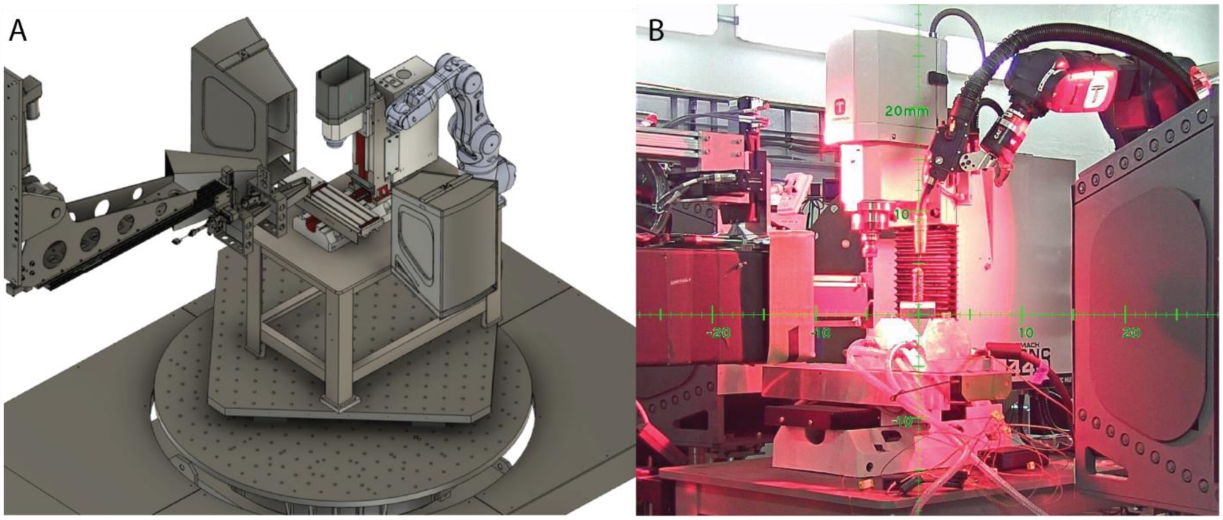

Supplementary Figure 1. Platform for additive manufacturing via Wire-Arc deposition integrated with VULCAN beamline at Oak Ridge National Lab's Spallation Neutron Source. (A) Mechanical design CAD of integration with VULCAN beamline (B) additive system setup on VULCAN beamline.

The robotic arm was used for motion control of the additive process. The maximum payload of the arm is 20 kg. Primary motion was achieved with motion of the first and third axis. A Tormach PCNC 440 CNC machining center was placed on the system table to serve as the primary experiment fixture point. While the CNC table was not used for relative motion during deposition, it provided gross alignment and fixturing. The CNC's range of motion is as follows: X-axis +/- 122mm from center, Y-axis +/- 76mm from center, Z-axis +254mm from table surface.

A Lincoln Electric R450, via metal inert gas (MIG) deposition, was leveraged for wire-arc additive manufacturing (WAAM). A PowerWave STT module and associated water chiller were installed as auxiliary components. Wire control was provided by 4R220 Autodrive WireFeeder. A Benzel WH500 22deg torch with co-axial wire feed and surrounding shielding gas were installed with the welder. All welding components were electrically isolated from other motion, sensing, and neutron diffraction components.

Fixturing to the CNC stage was accomplished with a custom-built aluminum riser (Supplementary Figure 2A), which was near-neutron transparent to avoid shadowing effects in the neutron diffraction detectors. An aluminum transfer plate (Supplementary Figure 2B) was

used to mount the mild steel build plates to the riser. Additionally, the riser was drilled for active cooling which was accomplished using forced compressed air. Active cooling was used at the end of depositing each section of material to reach room temperature for neutron mapping measurements more quickly. Supplementary Figure 2C shows infrared thermal data demonstrating that the peak temperature of the deposit reaches near room temperature in approximately 30 minutes while using the actively cooled substrate, compared to well over an hour without.

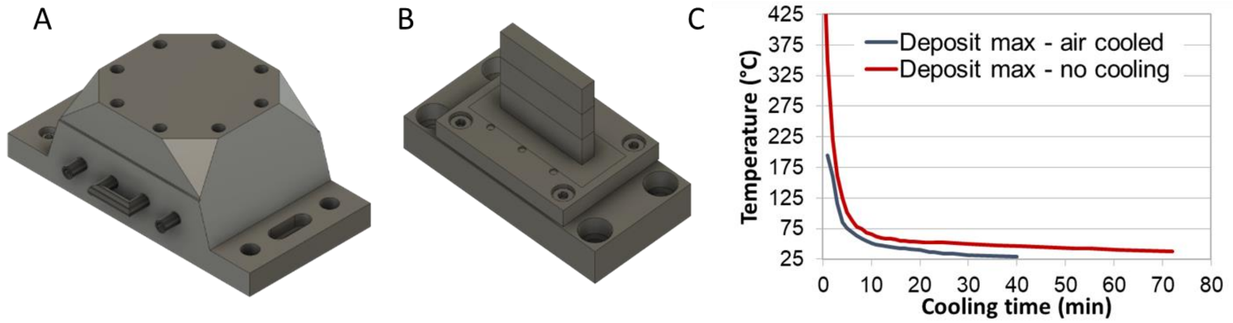

Supplementary Figure 2. (A) Aluminum riser with active air-cooled channels. (B) Steel substrate mounted on aluminum fixture plate (C) infrared thermal data showing differences in max temperature over time during cooling with and without the forced compressed air active in the substrate.

Toolpaths were planned with Openmind's HyperMILL and Roboris Eureka software packages. A standard longitudinal raster strategy was used for the base deposition toolpath. Welding wire with 1.1 mm diameter was used for all experiments. The measured composition of the LTT250 welding wire was the same as reported in previous publications[1,2] as shown in Supplementary Table 1. Deposition parameters for wire-arc additive manufacturing are given in Supplementary Table 2.

Supplementary Table 1. Measured chemical composition (wt.%) of the LTT250 welding wire.

C	Si	Mn	P	S	Cr	Ni	Mo	Ti	Al	B	N
0.05	0.3	0.7	0.01	0.01	11.9	5.4	0.01	0.006	0.01	0.001	0.006

Supplementary Table 2. Deposition parameters for AM via wire-arc welding of mild and LTT steels (compared in Figure 1).

**Parameter**	**Mild Steel**	**LTT**
Power	0.9 kW	1.1 kW
Wire Feed Rate	1905 mm/min	2794 min/min
Traverse Feed Rate	80 mm/min	80 mm/min
Wire Diameter	1.1 mm	1.1 mm
Argon Shielding Gas Flow Rate	15 cfm	15 cfm

While the same number of layers and the same scan pattern was used for both steels, the differences in material properties and process parameters resulted in different layer heights,

meaning that the height of each section and total build height were different, with the LTT sample tending to be taller. The location of neutron measurements were modified accordingly where appropriate.

Neutron Experimental Setup

Ex Situ Neutron Residual Stress Mapping

Ex situ residual strains (Figure 1D) were measured for the longitudinal and transverse directions at the High Intensity Diffractometer for Residual stress Analysis using a 3 x 3 x 3 mm^3 gauge volume. Strain distributions along the centerline of the mild steel and LTT walls were measured using 5 mm spacings. The interatomic spacing of the 211 BCC peak were extracted from the longitudinal and transverse diffraction data. Residual strains were assessed using the following relationship:

$$\varepsilon^{L,T}{}_{211} = \frac{d_{211}^{L,T}}{d_{211}^{O}} - 1 \quad (1)$$

where $d_{211}^{L,T}$ is the interatomic spacing of the (211) reflection for the Longitudinal or Transverse direction and d_{211}^{O} is the interatomic spacing for a stress-free reference. The stress-free reference was obtained by measuring diffraction data from LTT and mild steel coupons that stress relieved at 800°C for 8 hours.

Operando Data Reduction and Analysis

Diffraction data during WAAM were measured at VULCAN during deposition of LTT thin walls. VULCAN was configured using a 5 x 5 x 5 mm^3 gauge volume to isolate diffraction data from specific points along the build height. The gauge volume was positioned at the centerline of the wall. Event neutron data were measured continuously during deposition to capture the structural, lattice strain, and thermal expansion evolution. Measured event data were then binned in time and time-focused using a custom python script and the VDRIVEX[3] software package to reduce neutron events into 1 dimensional intensity vs d-spacing for each detector group. Both approaches used a common detector calibration that defined the detector offset for each pixel (obtained through a cross-correlation of data for a diamond sample). Data were reduced into 1 or 5 second intervals for visualization and analysis.

A 5 second integration time was used to reduce the noise in diffraction data used for Rietveld analysis. The GSAS software package[4,5] was used for Rietveld analysis to extract the evolution of martensite phase fraction during cooling. Prior to the analysis, a silicon standard was used to determine the instrument parameters. FCC (*Fm*-3*m*) and BCC (*Im*-3*m*) crystal structures were used to model the austenitic and martensitic volumes assuming that both structures had equivalent chemistries that did not vary. Supplementary Fig. 3 shows representative 1D diffraction patterns for the FCC and BCC crystal structures with characteristic peaks identified.

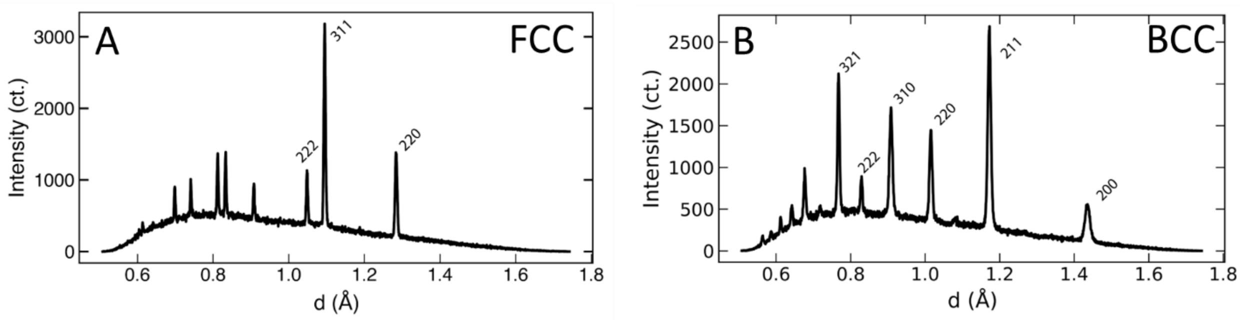

Supplementary Figure 3: Example 1D diffraction patterns for representative (A) FCC and (B) BCC phases.

The martensitic volume fraction was extracted using sequential refinement with results from each step used as the starting parameters for subsequent data. Parameters refined include scale parameter, 5th order background, Martensitic volume fraction, hydrostatic strain (both phases), isotropic microstrain (both phases) and crystallite size (both phases). The phase fraction of BCC calculated in this way is shown in Supplementary Figure 4 during the cooling period following deposition of each of the three build sections. Notably, the transformation rate is fastest following the first section for which the point being measured is closest to the actively cooled aluminum riser. Subsequent points at further distances from the base plate have longer cooling times and slower phase transformation rates (see temperature data in Figure 3).

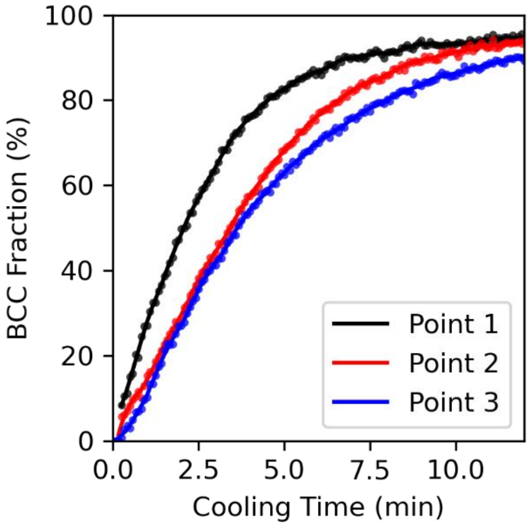

Supplementary Figure 4. Phase fraction of BCC as a function time during cooling following deposition of each build section.

Time-dependent interatomic spacing for observed diffraction peaks of ferrite, austenite and martensite were extracted from the operando data using the GSAS single peak fitting approach implemented in VDRIVEX. It is possible to calculate the time-resolved elastic lattice strains by estimating the effective strain free reference as a function of temperature and thermal expansion based on the IR data. However, because the IR data is at different temporal and spatial

resolutions and is further a surface measurement, compared to the volumetric neutron diffraction data. As a result, this method, although technically sound, tends to produce noisy results. Instead, we choose to use a technique that relies only on the neutron data for strain calculation, and leverages the IR data for error estimation. The time dependent lattice strain was calculated according to Equation 4 in the main text based on the difference in lattice peak shift for either the longitudinal direction ($\widehat{Q_1}$ from detector 1) or transverse direction ($\widehat{Q_2}$ from detector 2) and the high-angle direction ($\widehat{Q_3}$ from detector 3). This approach yields an accurate measurement of the normal elastic lattice strains in the transverse and longitudinal direction only when the elastic lattice strain in detector 3 may be considered negligible by comparison. This assumption is equivalent to saying that the peak shift in detector 3 is only a function of temperature. In this case, the temperature may be calculated from the neutron diffraction (ND) signal in detector 3 using Equation 5 in the main text. This determination of temperature depends on accurate knowledge of the strain-free, room temperature reference, $d_{hkl}^{\widehat{Q_i},0}$. For BCC, the reference value was measured directly from a well-annealed sample. For FCC, it was necessary to establish an effective reference value, which could not be measured directly. In this case, the temperature was calculated for FCC and BCC for neutron diffraction times in which both phases existed simultaneously and using the CTE value for each phase. It was assumed that the temperature within the 5 mm neutron voxel was uniform during these times, and the Nelder-Mead algorithm[6] was used to optimize an effective $d_{hkl}^{\widehat{Q_i},0}$ value for each detector bank by minimizing the predicted temperature difference between the phases. This approach was applied for the (311) FCC reflections, and the results are reported for each detector bank in Supplementary Table 3. Note that these values are not equivalent to what might be measured for the retained austenite at room temperature, as the CTE is considered constant for each phase across all temperatures. Therefore, the FCC temperature calculated using this technique are only valid for temperature above the two-phase region near M_s.

Supplementary Table 3. Optimized reference values for FCC for each detector bank based on a temperature comparison with BCC.

**Detector Bank**	**Optimized d_{311}^0**
1	1.0799
2	1.0800
3	1.0800

Using the measured and optimized d_{hkl}^0 values, as well as corresponding CTE values for each phase, it was possible to reconstruct an estimated temperature history based on the time-resolved neutron diffraction data collected in detector bank 3. This data is not used directly for the calculation of the elastic lattice strains, as that calculation may be performed by simply taking the difference in peak location as given by Equation 5. However, that calculation is only valid if the strains in detector 3 are small. If this assumption is true, then the temperature estimated in this way will also be accurate and may be judged as so relative to an independent dataset. In this case, we may use the IR temperature data to evaluate the validity of this assumption, and to quantify the error of the elastic lattice strain measurements. For this purpose, it was first

necessary to align the ND and IR data. This alignment was performed by computing the temperature in FCC during the cooling period after building of that section, and again using a Nelder-Mead algorithm to optimize a time-offset for the IR data that minimized the difference between temperature signals for each case. The error in the time dependent elastic strain determination is then given by multiplying the absolute value of the difference between the temperature signals by the coefficient of thermal expansion, α:

$$err(t) = \alpha|T_{IR}(t) - T_{ND}(t)| \quad (2)$$

where T_{IR} is the temperature given by the IR measurement, and T_{ND} is the temperature calculated form the neutron diffraction data. The CTE for each phase was based on the dilatometry data (Fig. 1), and assumed to be constant for temperature. CTE for FCC is valid between M_s and approximately 1000°C, and for BCC, between room temperature and A_s.

Residual strains for the longitudinal and transverse directions were also measured after deposition of each wall section using a 5 x 5 x 5 mm^3 gauge volume. Diffraction data were measured using a 5 x 5 mm (x, y) grid. The interatomic spacings of the (211) BCC peak were extracted from the longitudinal and transverse diffraction data. Residual strains were assessed using equation S1. The stress-free references were obtained by measuring diffraction data from LTT and mild steel coupons that stress relieved at 800°C for 8 hours.

Additional Neutron Data

Figure S5 shows a summary of all the time resolved diffraction data collected from the operando AM experiments. Note that data was collected at point 1 during fabrication of all three sections, but was not presented in the main text for building of sections 2 and 3. The analysis of the operando data for point 1 during fabrication of the upper two sections is shown in Figure S6. The same alignment procedure was used to couple the IR and neutron diffraction (ND) data (Supplementary Figure 6A), except that, because only BCC was present and this point did not transform back to FCC, the temperature from the ND data was estimated using the (211) reflection in detector bank 2. The IR data was then interpolated and used to calculate time-dependent reference lattice spacings. During fabrication of section 2, the longitudinal strain at point 1 becomes highly compressive, caused by the formation of an austenite reversion zone above. During cooling the strain begins to decrease in magnitude, eventually approaching a mildly compressive state as shown by the map in Figure 4. During fabrication of section 3, the phase transformations and high thermal gradients present during processing are far enough away from point 1 that they do not strongly affect the elastic lattice strains during printing.

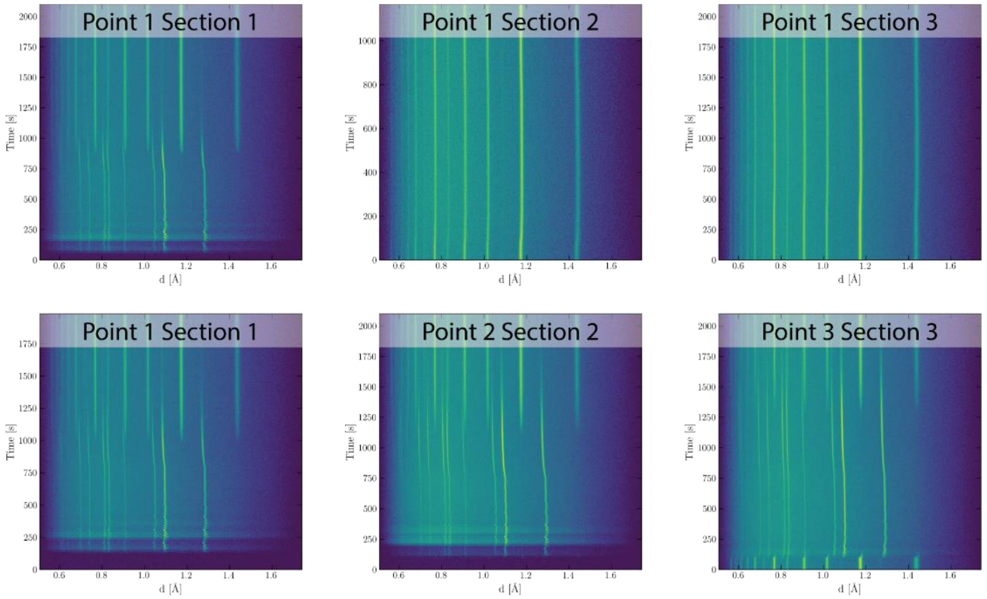

Supplementary Figure 5. Summary of time resolved neutron diffraction data for the LTT steel builds taken during fabrication of each section.

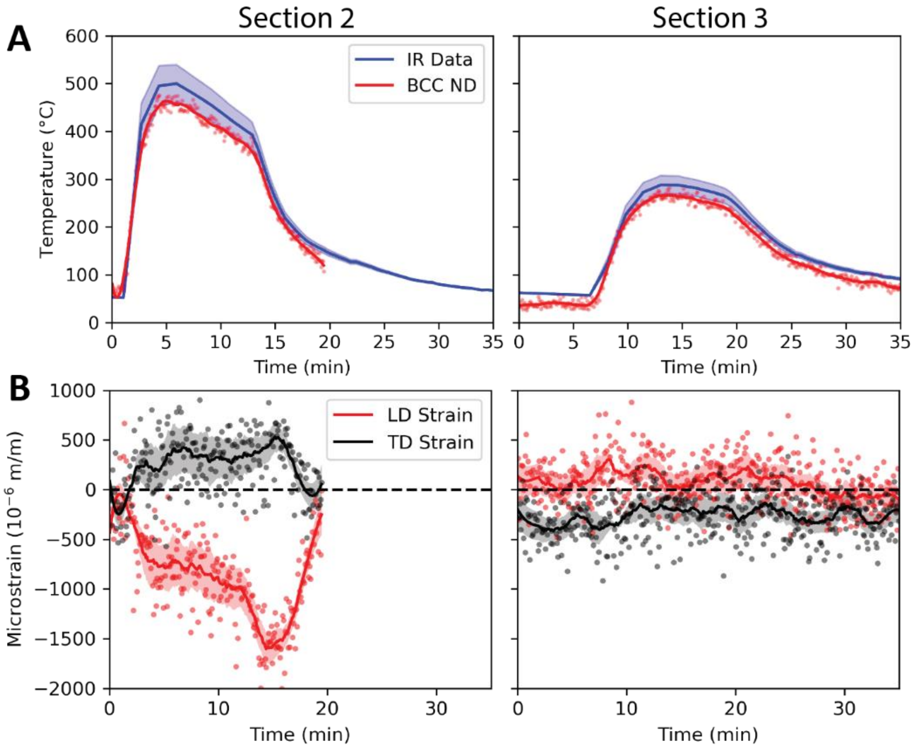

Supplementary Figure 6. Analysis of time-dependent neutron diffraction data for point 1 during sections 2 and 3, showing (A) the alignment of the neutron data based on an estimated temperature signal for the BCC 211 peak in detector 2, and (B) the calculated lattice strains as a function of time using the aligned IR data for calculating a time-dependent reference lattice spacing.

Infrared Imaging Experimental Setup

Two infrared cameras were positioned to measure component surface temperatures (Supplementary Figure 7). The infrared cameras used were FLIR A700 uncooled microbolometers with sensor resolution of 640x480 pixels, 30 Hz sample rate, a 17 mm focal length lens, and spectral sensitivity in the 8-14 µm range. The cameras were assembled with a protective window and a five-slot ZWO programmable filter wheel (Supplementary Fig. 7). Thorlabs series FBXX000-500 bandpass filters were used and are listed in Table S4. All filters had a nominal FWHF transmission window of 0.5 µm and were mounted at 10 degrees off the optical axis to remove secondary camera reflections.

The filter wheels were swept through each combination immediately after deposition was completed for each layer, and periodically every 60 seconds afterward if deposition had not resumed. The spectral IR signal for the 10 µm wavelength used for the analysis was only collected in the interpass wait times.

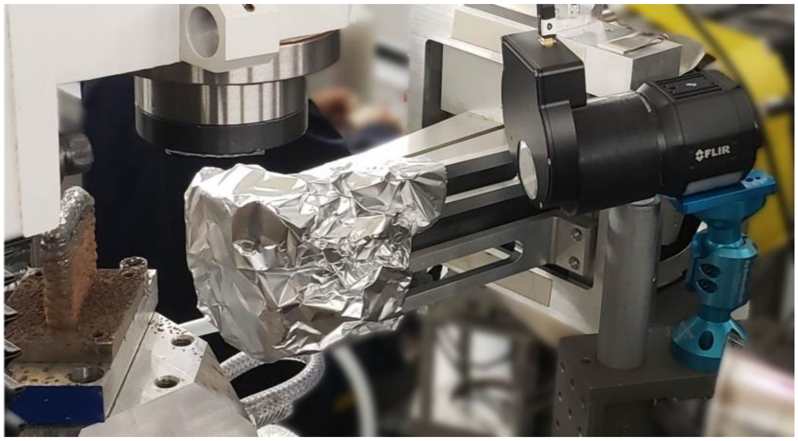

Supplementary Figure 7. IR camera from bank 2 mounted adjacent to shielded neutron slit viewing surface of deposited LTT wall.

Supplementary Table 4. Infrared filters used for multispectral imaging.

**IR camera 1 CWL**	**IR camera 2 CWL**
Empty slot	Empty slot
8.5 µm	8 µm
9 µm	9 µm
9.5 µm	10 µm
10 µm	11 µm

Thermal sensor spectral response for each filter wavelength was calibrated against a NIST traceable Mikron M305 high temperature blackbody furnace with cavity emissivity of 0.995 and measurement uncertainty of ±0.2% of reading ±1°C. Sensor response was fit with the Plank blackbody equation[7]:

$$B_\lambda(T) = \varepsilon \cdot E_\lambda(T) = \frac{2hc^2}{\lambda^5} \frac{1}{e^{hc/k_b \lambda T} - 1}, \tag{3}$$

where B_λ is greybody spectral radiance, E_λ is blackbody spectral radiance, h is the Plank constant, c is the speed of light, and k_b is the Boltzmann constant. Corrections were included for sensor dark noise, filter CWL shift from optical tilt, and a pixelwise correction for secondary filter reflection and transmission:

$$S = \varepsilon_{obj}\, C \int T_\lambda Q_\lambda B_\lambda(T_{obj})\, d\lambda + \varepsilon_{ref} C \int (1 - T_\lambda)\, Q_\lambda\, B_\lambda(T_{ref})\, d\lambda + DN, \tag{4}$$

where S is the signal sensor response, C is a calibration parameter for the combined throughput response that includes sensor ADC and gain, ε_{obj} and ε_{ref} are the target and reflected object emissivity, T_{obj} and T_{ref} are target and reflected object absolute temperature, T_λ is spectral transmittance of the filter, Q_λ is the sensor spectral quantum efficiency, and DN is an offset for sensor dark noise for a given sensor temperature. Signal sensor response is calculated with numerical integration and a lookup table generated for rapid real-time temperature calculation.

Real emissivity of the deposited oxidized material was calibrated using a low mass K-type thermocouple in thermal contact near the sample surface, which was heated to 80°C and allowed to stabilize. For printed material, oxidized LTT normal emissivity in the sensor sensitivity band was measured to be 0.95, implying optically thick oxidation[8–10]. A constant emissivity value was assumed which discards the effects of temperature dependence of emissivity, oxide growth during deposition, partial oxide spallation from CTE mismatch, and surface roughness.

Supplementary Note 2: Materials Characterization

Characterization Methodology

The LTT build was removed from the build plate and sectioned into two halves which were then prepared using standard metallographic techniques, including polishing 1-micron surface finish and final vibratory polish in colloidal silica. Electron backscatter diffraction (EBSD) was performed using a Zeiss Crossbeam 550 dual beam FIB/SEM with Oxford XMax EBSD detector. EBSD mosaics were acquired across the entire length of the sample using a step size of 2 microns. EBSD data were post-processed using MTEX, an open-source MATLAB code, to extract inverse pole figures and Kernel Average Misorientation (KAM) maps. Parent austenite grains were reconstructed from the raw EBSD data of the martensite structure using an iterative orientation relationship (OR) determination and Markov clustering[11,12]. Hardness measurements were conducted across using a LECO AMH 55 automatic hardness tester with a load of 1 kgf and a dwell time of 15 seconds. A 3x150 grid with a grid spacing of 500x500 µm was used, and the data presented here is the average of 3 points.

LTT Steel Characterization Results

Pole figures for both martensite and austenite were extracted from high and low KAM regions. There is no clear texture or preferred orientation for either phase. Comparing the $(111)_\gamma$ and $(110)_\alpha$ pole figures, the Kurdjumov-Sachs (K-S) orientation relationship is obeyed between parent austenite and martensite. The variant ID and their normalized frequency were extracted from the EBSD data and plotted as histograms. In both regions, no preferred variant selection was observed.

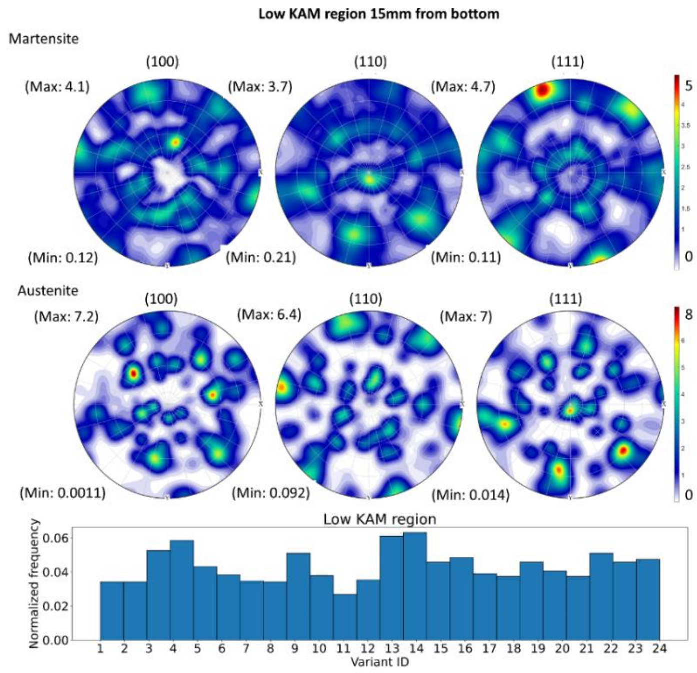

Supplementary Figure 8. Pole figures of the BCC martensite and reconstructed FCC austenite phases from the low KAM region along with a histogram showing no strong variant selection.

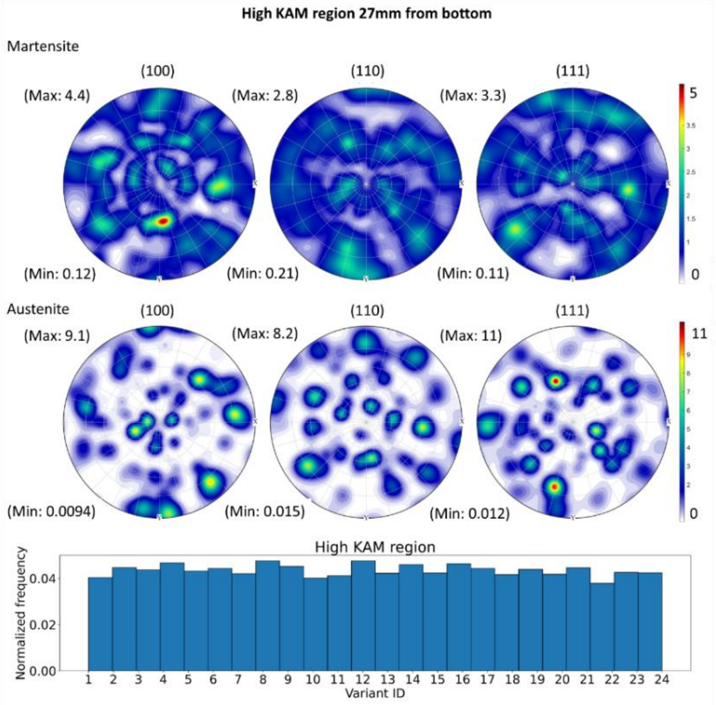

Supplementary Figure 9. Pole figures of the BCC martensite and reconstructed FCC austenite phases from the low KAM region along with a histogram showing no strong variant selection.

Supplementary Note 3: Simulation Methodology and Results

Thermomechanical Model

A three-dimensional physics-based FE model was developed using the Abaqus 2020 AM Module and expanded to incorporate solid-state phase transformations; both approaches are described in the literature[13–15]. The part and substrate (Supplementary Figure 10) was discretized into 113,220 8-node hexahedral 1 mm^3 elements for heat transfer (DC3D8) and stress (C3D8) analysis. In the heat transfer analysis, a convection coefficient of 60 W/m^2°C was applied at the bottom boundary during printing and increased to 1000 W/m^2°C during cooling to account for forced convection. The sample and substrate surfaces were given an emissivity of 0.3 for radiative heat transfer. During stress analysis, the bottom surface of the substrate was constrained. The event series required for progressive element activation and heat addition was generated from the toolpath obtained from the robotic system.

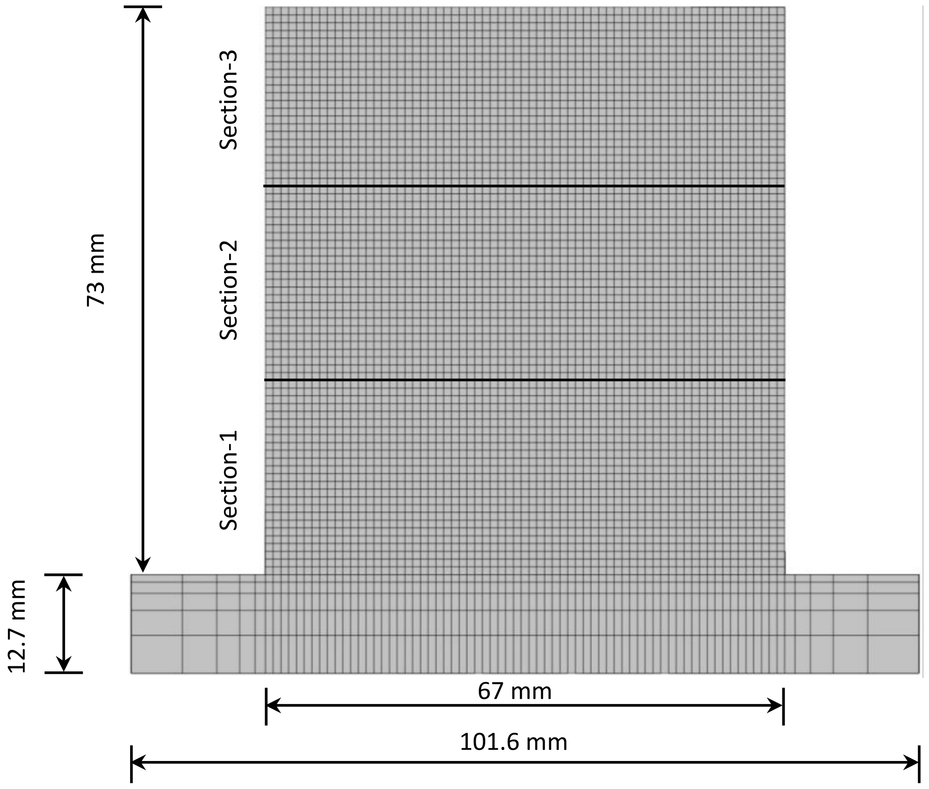

Supplementary Figure 10. Schematic with model dimension and mesh structure.

Temperature-dependent material properties were adopted for both thermal and stress analysis. Specific heat, thermal expansion coefficient, and yield strength were obtained from previous experiments[2]. Thermal conductivity, density, and plastic properties were obtained from JMAT pro and the literature[16,17]. The thermal expansion during stress analysis was employed using the UEXPAN subroutine for phase dependent thermal expansion and to include transformation induced plasticity (TRIP) during the FCC to BCC transformation.

Phase transformation model

Phase transformations between FCC and BCC were integrated such that temperature changes in the elements lead to phase transformations based on prescribed transformation conditions. The BCC phase fraction during cooling was based on an empirical relationship[18]. The K-M equation below gives the martensite fraction, f_m, depending on the temperature and the empirical constant γ is calibrated for the present LTT steel composition using the BCC fraction formation obtained from neutron measurements:

$$f_m = f_m(\theta_0) + (f_a(\theta_0) - f_{ar})(1 - \exp[-\gamma(M_s - \theta)]), \tag{5}$$

where θ_0 is the temperature at the beginning of the cooling cycle, M_s is the martensitic start temperature, f_a is fraction of austinite, f_{ar} is the retained austinite.

Validation of The Temperature Profile

Accurate prediction of the phase transformation and its effect on the residual stresses are dependent on transient thermal profiles. Thermal profiles in the middle of the part 5 mm above the substrate during the deposition of section 1, 2, and 3 of the part were compared with the temperature calculated from neutron diffraction. The simulation temperature was taken to be the average over the elements occupying the same volume as the 5 mm diffracted voxel. The temperature profiles agree well with the temperature estimated from the neutron data (Supplementary Figure 11). The calculated temperature profiles were used for phase transformation and residual stress calculations.

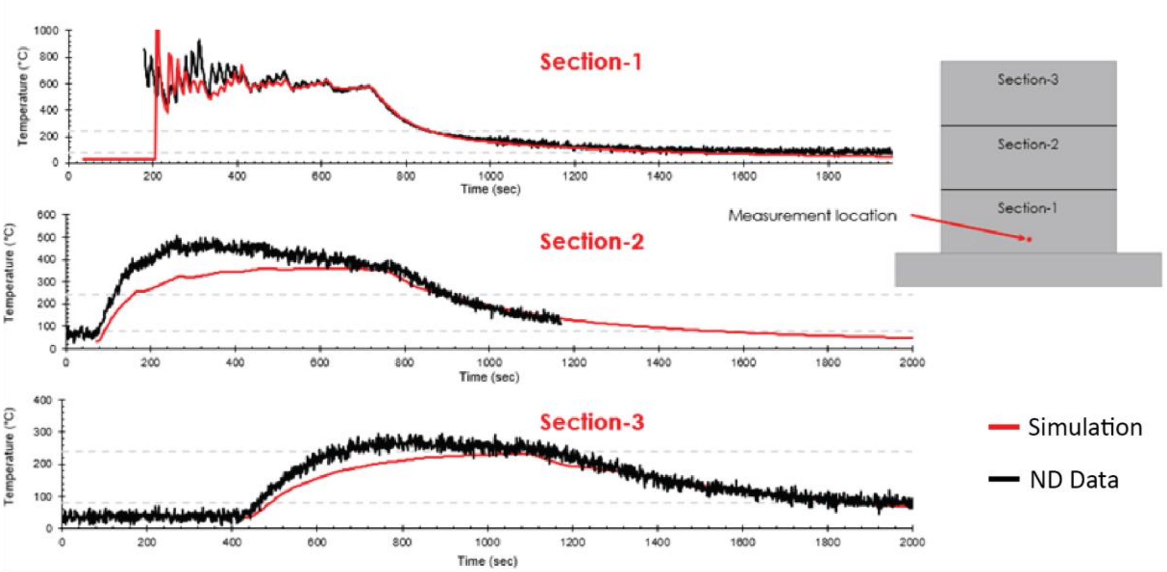

Supplementary Figure 11. Comparison of model predicted temperature profiles averaged across a 5 mm cubic voxel to experimentally measured temperature from the neutron diffraction peak shift as described by Equation 1.

Validation of phase transformation

After the calibration of the thermal profile, it was applied to a single element to determine the phase fraction and calibrate the K-M equation empirical coefficient γ which is dependent on material. The plot in Supplementary Figure 12 shows the comparison of the FCC fraction from Rietveld analysis of the ND data with the calculated fraction from the FE model. The FE model agrees well with the trend and profile of the phase transformation profile.

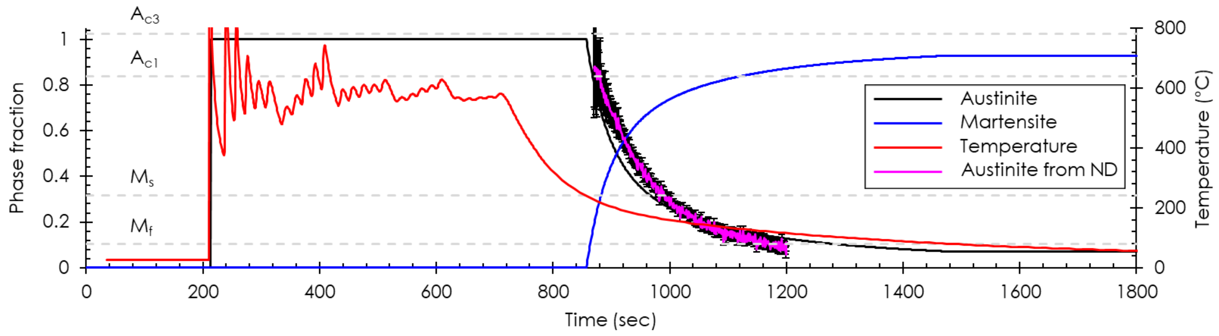

Supplementary Figure 12. Model temperature and associated phase fraction prediction validated against phase fraction data determined from operando neutron diffraction data.

Elastic Strain Predictions

Supplementary Figure 13 summarizes the predicted longitudinal elastic strain distribution in response to the temperature distribution and phase transformation behavior during and after deposition of section 2. The simulation results show several distinct steps in the elastic strain development. During deposition of section 2, because the heat from deposition converts some BCC back to FCC in section 1, a boundary is formed between the FCC and BCC phases below the interface between the first two sections. In this phase, with a predominantly vertical thermal gradient (along the z-direction), the expansion of the newly deposited and converted FCC with higher CTE tends to produce a tensile longitudinal strain field above the FCC/BCC boundary, and a compressive strain field in the BCC below. Initially upon cooling, there is no phase change during the time which it takes material newly converted to FCC to traverse the whole temperature range up and back down to the M_s temperature (240°C). During this cooling period, the FCC austenite contracts more severely due to a higher CTE, continuing the development of tensile strains with counter compressive strains in the BCC martensite below. Eventually, the FCC region cools below the M_s to induce the transformation to BCC. The volume expansion from FCC to BCC caused a strain reversal near the FCC/BCC interface (Figure 6A). The newly formed BCC experiences compressive strains while counter tensile strains developed in the previously existing BCC, which is needed to maintain mechanical equilibrium. As the phase transformation completes, the compressive strains in BCC are strengthened above the boundary and the counter tensile stresses are intensified in section 1 below and with lower tensile strains near the top of section 2. Even though the phase transformation occurs throughout section 2, compressive strains are not observed in all regions of section 2 due to the larger magnitude of thermal strain compared to volumetric expansion from the FCC to BCC phase transformation. During the phase transformation process, at any location if the thermal strain due to temperature change is higher than the strain induced by volumetric expansion of the BCC, tensile strains were observed. Due to the Poisson effect, the transverse strains (Supplementary Figure 14) generally

show the opposite trends as the longitudinal strains with respect to tension/compression, but with a lower magnitude.

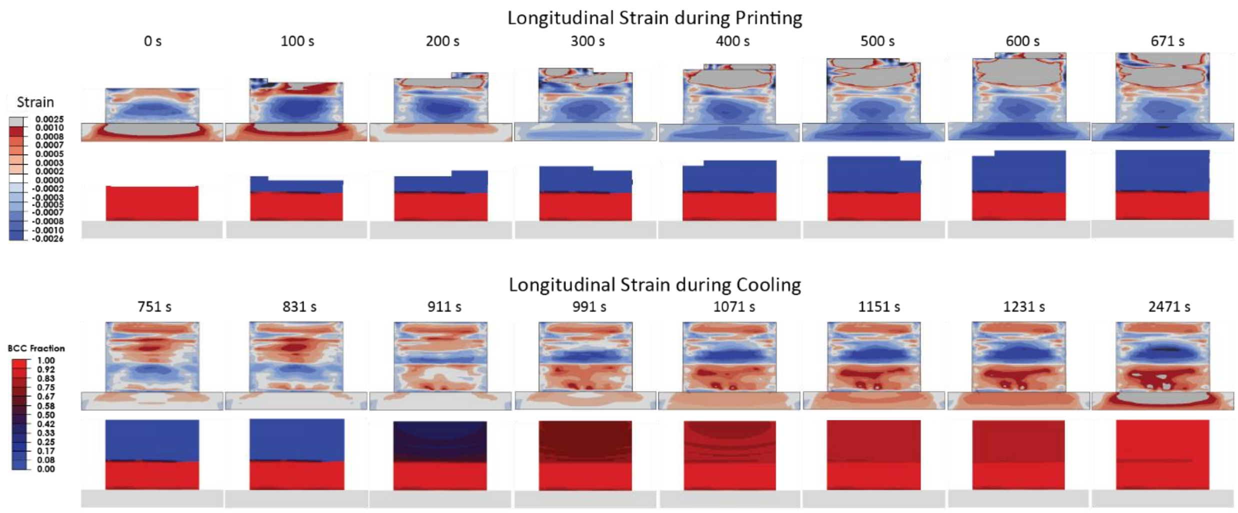

Supplementary Figure 13. Longitudinal strain evolution and BCC fraction during fabrication and cooling of section 2 as precited by the coupled thermomechanical-phase transformation model.

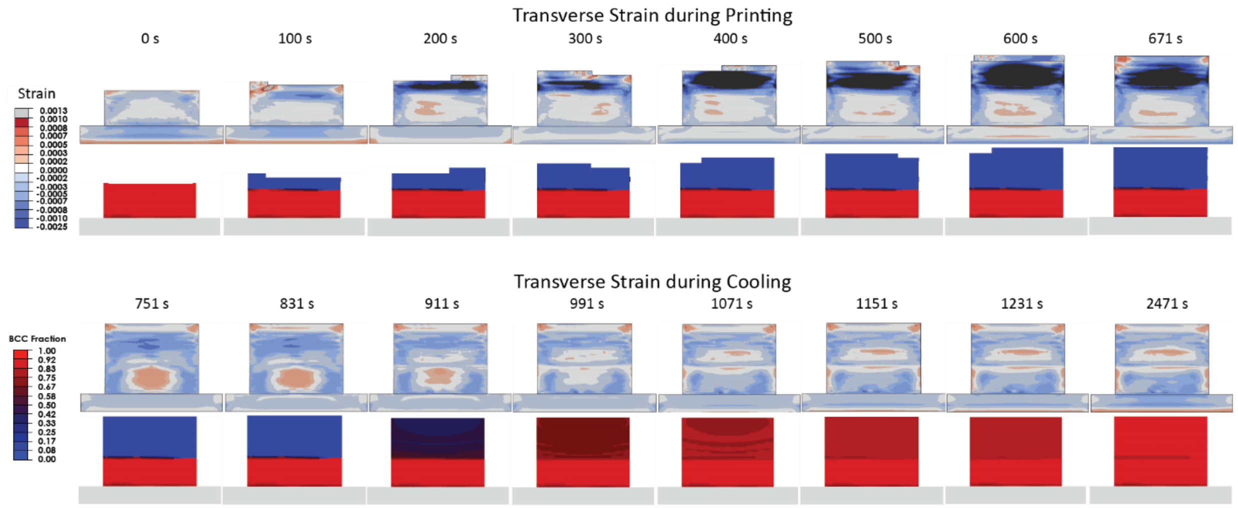

Supplementary Figure 14. Transverse strain evolution and BCC fraction during fabrication and cooling of section 2 as precited by the coupled thermomechanical-phase transformation model.

The time-resolved neutron diffraction analysis of the elastic lattice strains depends on the assumption that the strain measured in detector 3 is only a function of temperature. One potentially error in this assumption is due to the Poisson effect from strains in the vertical direction, which are not directly measurable using the detector configuration at VULCAN.

Therefore, we evaluate the quality of this assumption by considering the results of the thermomechanical model. Supplementary Figure 15 shows a comparison of the longitudinal, transverse, and vertical strains for two selected points during and following fabrication of section 2. Overall, the vertical strains are found to be relatively small compared to the longitudinal strains, but roughly the same magnitude as the transverse strains. However, because these effects are only important through the Poisson effect, for $\nu = 0.3$, the effect of vertical strains is roughly similar to or smaller than the transverse strain. More importantly, this effect is expected to be similar for all in-plane directions. Therefore, when calculating time-resolved elastic lattice strains as shown in Equation 4, the effect cancels out. The vertical strain will only influence the calculation of the time-resolved in-plane stress if the Poisson effect is anisotropic as a result of a strong crystallographic texture. As shown in Fig. S8 and S9, the texture in this case is not strong, and the difference in Poisson effect between either detector 1 or 2 and detector 3 is expected to be strong. Finally, because of the approach to error estimation against the IR data, this effect is accounted for in the plotting of the error bounds in Figure 4. Overall, the effect of the vertical strain is not expected to be significant or dramatically change any of the trends or subsequent conclusions reported here.

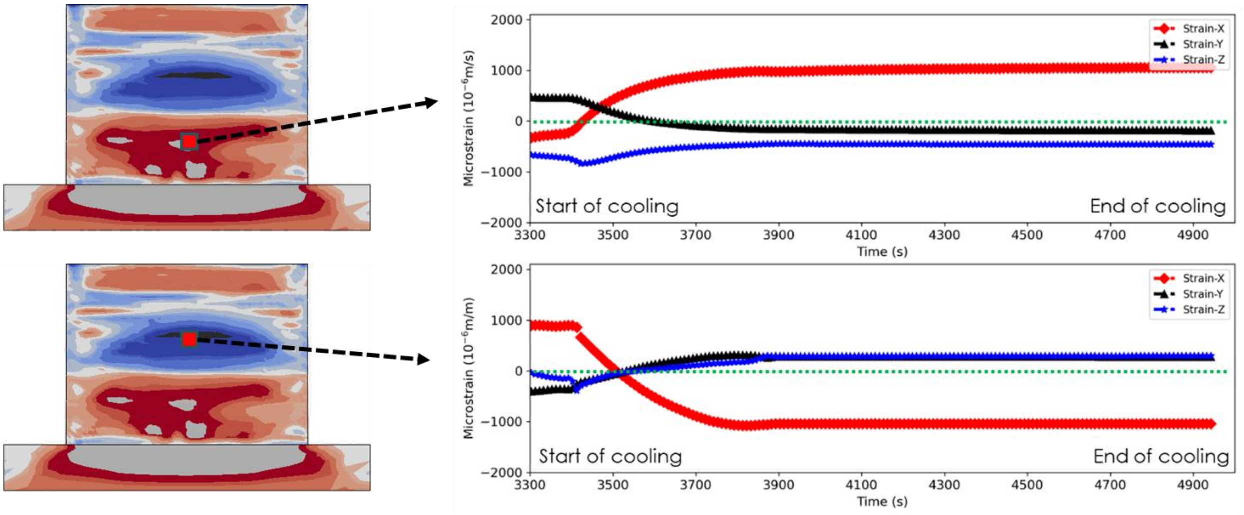

Supplementary Figure 15: Comparison of longitudinal, transverse, and vertical strain components during and after fabrication of section 2.

Supplementary References

1. Sridharan, N. *et al.* Consumable development to tailor residual stress in parts fabricated using directed energy deposition processes. *Addit. Manuf.* **39**, 101837 (2021).
2. Tang, W. *et al.* Characteristics of Wire Arc Additively Manufactured Low Transformation Temperature Steel. *Prep.* 1–23.

3. Reduction, D. & Diffraction, M. N. *VDRIVE Data Reduction and Interactive Visualization softwarE for Event*. (2012).
4. Larson, A., Dreele, R. & Toby, B. General structure analysis system-GSAS/EXPGUI. (1994).
5. Toby, B. H. EXPGUI, a graphical user interface for GSA_S [-J]. *Joumal Appl. Crystallogr.* **34**, 210–213 (2001).
6. Nelder, J. A. & Mead, R. A simplex method for function minimization. *Comput. J.* **7**, 308–313 (1965).
7. Planck, M. *The theory of heat radiation*. (P. Blakiston's Son & Co., 1914).
8. del Campo, L., Pérez-Sáez, R. B. & Tello, M. J. Iron oxidation kinetics study by using infrared spectral emissivity measurements below 570 °C. *Corros. Sci.* **50**, 194–199 (2008).
9. Fu, T., Tan, P. & Zhong, M. Experimental research on the influence of surface conditions on the total hemispherical emissivity of iron-based alloys. *Exp. Therm. Fluid Sci.* **40**, 159–167 (2012).
10. Wen, C. Da. Investigation of steel emissivity behaviors: Examination of Multispectral Radiation Thermometry (MRT) emissivity models. *Int. J. Heat Mass Transf.* **53**, 2035–2043 (2010).
11. Nyyssönen, T. Quenching and Partitioning of High-Aluminum Steels. 100.
12. Nyyssönen, T., Isakov, M., Peura, P. & Kuokkala, V.-T. Iterative Determination of the Orientation Relationship Between Austenite and Martensite from a Large Amount of Grain Pair Misorientations. *Metall. Mater. Trans. A* **47**, 2587–2590 (2016).
13. Lee, Y. S. *et al.* Role of scan strategies on thermal gradient and solidification rate in electron beam powder bed fusion. *Addit. Manuf.* **22**, 516–527 (2018).

14. Nycz, A. *et al.* Effective residual stress prediction validated with neutron diffraction method for metal large-scale additive manufacturing. *Mater. Des.* **205**, 109751 (2021).

15. Zhang, Q. *et al.* A metallurgical phase transformation framework applied to SLM additive manufacturing processes. *Mater. Des.* **166**, (2019).

16. Saunders, N., Guo, Z., Li, X., Miodownik, A. P. & Schillé, J. P. Using JMatPro to model materials properties and behavior. *JOM* **55**, 60–65 (2003).

17. Jiang, W. *et al.* Effects of low-temperature transformation and transformation-induced plasticity on weld residual stresses: Numerical study and neutron diffraction measurement. *Mater. Des.* **147**, 65–79 (2018).

18. Koistinen, D. P. A general equation prescribing the extent of the austenite-martensite transformation in pure iron-carbon alloys and plain carbon steels. *Acta Metall.* **7**, 59–60 (1959).
